# An OWA Distance-Based, Single-Valued Neutrosophic Linguistic TOPSIS Approach for Green Supplier Evaluation and Selection in Low-Carbon Supply Chains

**DOI:** 10.3390/ijerph15071439

**Published:** 2018-07-08

**Authors:** Ji Chen, Shouzhen Zeng, Chonghui Zhang

**Affiliations:** 1 College of Statistics and Mathematics, Zhejiang Gongshang University, Hangzhou 310018, China; chenji810404@zjgsu.edu.cn (J.C.); zhangch1988@zjgsu.edu.cn (C.Z.); 2 School of Business, Ningbo University, Ningbo 315211, China

**Keywords:** single-valued neutrosophic linguistic set, OWA operator, TOPSIS, multiple attribute decision-making, green supplier selection

## Abstract

This paper presents a technique based on the ordered weighted averaging (OWA) distance for the single-valued neutrosophic linguistic (SVNL) technique for order preference by similarity to an ideal solution (TOPSIS). First, the inadequacies of the existing SVNL TOPSIS are analyzed in detail. Second, a SVNL OWA distance (SVNLOWAD) measure is presented, and based on this, a modified TOPSIS, termed the SVNLOWAD-TOPSIS, is developed for multiple attribute decision-making problems with SVNL information. Third, a revised relative coefficient is proposed to rank potential alternatives. Finally, a numerical example concerning green supplier selection in low-carbon supply chains is introduced to demonstrate the effectiveness of the model.

## 1. Introduction

Due to growing awareness of the need to protect the environment and address global warming, low-carbon supply chains have become a leading research topic in previous decades [1,2,3]. Identifying a suitable green supplier is one of the most crucial activities to achieving a high efficiency, low-carbon supply chain to play a critical role in environmental sustainability and carbon emissions reduction [4,5]. The process of supplier selection is generally considered to be a kind of multiple attribute decision-making (MADM) problem, since numerous and varied attributes should be embedded and assessed in the decision process [6,7,8,9,10]. In the literature, types of MADM methods concerning supplier selection have been put forward; the guiding hypothesis for utilizing these approaches is to assume that the attribute information is precisely known and can be accurately assessed [11,12,13,14,15]. However, the increasing complexity of objects makes it difficult for decision makers (DMs) to perfectly assess preference information about their attributes during the selection process. Therefore, imprecise and uncertain evaluations often appear in practical MADM problems. Handling uncertainties or imprecision effectively under these situations has become a crucial issue in MADM analysis. Consequently, several tools for describing such uncertainties or imprecision information have been proposed, one of which is the single-valued neutrosophic set (SVNS) introduced by Smarandache [16] and Wang et al. [17]. As a new and useful extension of fuzzy sets, the SVNS is characterized by the degree of its truth-member, the degree of its indeterminacy-member and the degree of its falsity-member. In comparison to other fuzzy tools, such as the intuitionistic fuzzy set (IFS) [18] and the Pythagorean fuzzy set (PFS) [19,20], the SVNS appears to be more effective for handling uncertain information, as it can address indeterminate information that IFS and PFS cannot. Following this recent research trend, SVNS theory has been viewed as being suitable for dealing with MADM problems under uncertain and complex situations. Ye [21] developed a weighted correlation coefficient for SVNS and studied its application in MADM problems. Ye [22] also studied its application by using single-valued neutrosophic (SVN) cross-entropy. Biswas et al. [23] introduced a gray relational analysis approach for addressing a MADM problem with SVN information. Zavadskas et al. [24] analyzed the usefulness of a classic weighted aggregated sum product assessment (WASPAS) method in SVNL MADM problems. Ye [25] extended some classic operators to aggregate SVN information. Meanwhile, Ye [26,27] developed some measures of similarity between SVNSs and explored their application in clustering analysis and decision-making problems. Huang [28] introduced a distance measure method for MADM in an SVN environment. Liu [29] developed some Archimedean t-conorm and t-norm operators to aggregate SVN information in MADM. Li et al. [30] proposed several SVN Heronian mean operators and applied them in MADM. Pramanik et al. [31] introduced a series of hybrid vector similarity measures for SVNSs in MADM analyses. Peng et al. [32] extended the outranking method to SVN situations and applied it to MADM problems.

In contrast to the research mentioned above, in numerous MADM problems, the evaluations of attributes cannot be clearly evaluated quantitatively, but may be evaluated qualitatively, and it may be easier to address these problems using linguistic information. For instance, people like to use terms like “bad”, “medium” or “good” to describe the “comfort” or “design” of a vehicle. Hence, the fuzzy linguistic method [33] presents an attractive way of handling this uncertainty in terms of linguistic variables and has been applied widely in the MADM field. Recently, Ye [34] proposed the single-valued neutrosophic linguistic set (SVNLS), whose basic element is the single-valued neutrosophic linguistic number (SVNLN). By integrating the advantages of SVNS and linguistic terms, the SVNLS can eliminate the drawbacks of each of the, and has been found to be able to accommodate a higher degree of uncertainty of subjective assessments. With SVNLS, Wang et al. [35] investigated an SVNL aggregation operator using the generalized Maclaurin symmetric mean method. Luo et al. [36] developed a distance-based model for a simplified neutrosophic linguistic MADM problem. Tian et al. [37] proposed the QUALIFLEX method to address the selection of problems concerning green products with SVNL information. Wu et al. [38] extended the SVNLS to a two-tuple situation, and studied some of its operational rules and applications to MADM analyses.

Among the various MADM approaches, the technique for order preference by similarity to an ideal solution (TOPSIS) method [39] continues to perform effectively in many applications. The main idea of the TOPSIS approach is the establishment of ideal solutions and the subsequent ordering of alternatives by using distances from the ideal solutions. Accordingly, TOPSIS has been utilized successfully to solve various MADM problems—its concept is rational and easy to grasp and compute. Information about the evaluation of alternatives provided by DMs is given precise values in the traditional TOPSIS approach. Over the past several decades, the TOPSIS approach has been used to handle MADM problems in different kinds of fuzzy situations, such as in fuzzy number contexts [40], IFS contexts [41,42], linguistic sets [43] and Pythagorean fuzzy environments [20]. Biswas et al. [44] introduced the use of the TOPSIS model to deal with SVNS and subsequently, applied it in a MADM problem. Khorram et al. [45] studied a SVNL TOPSIS with multiple DMs. Peng and Dai [46] explored the SVNL TOPSIS method by using a new scoring function and similarity measure. Ye [34] proposed an extension of the TOPSIS approach to deal with an SVNLS MADM problem.

Given the detailed analyses in the research referred to above, it is clear that all of the previously mentioned TOPSIS approaches share a same problem that needs to be solved—they fail to address the DMs’ attitudes during the decision-making process. As a consequence of this, one cannot present a general model for diverse aggregation processes that is related to the DMs’ interests. The latter issue is especially important whenever variation in degrees of optimism and pessimism needs are to be considered. To circumvent this drawback, this paper develops a revised SVNLS TOPSIS technique and explores its validity and applicability in MADM problems concerning green supplier selection.

The remainder of this paper is structured as follows. Preliminaries to the SVNLS are discussed in Section 2. A SVNL ordered weighted averaging distance (SVNLOWAD) measure is introduced in Section 3, and a modified TOPSIS method involving SVNL information based on the proposed distance measure, called SVNLOWAD-TOPSIS, is introduced. Section 4 presents a numerical example concerning green supplier selection to show the application and effectiveness of this revised TOPSIS model. Finally, in Section 5, we discuss our primary conclusions.

## 2. Preliminaries

This section briefly summarizes some key concepts of SVNS and SVNLS, including definitions, operational laws and distance measures.

### 2.1. The Single-Valued Neutrosophic Set

**Definition** **1** [17]. *Let* x *be an element in a finite set,* X*. A single-valued neutrosophic set (SVNS),* P*, in* X *can be defined as in (1):*

(1) $$P=\left\{\langle x,T_{P}(x),I_{P}(x),F_{P}(x)\rangle |x\in X\right\},$$

*where the truth-membership function,* $T_{P}(x)$*, the indeterminacy-membership function,* $I_{P}(x)$*, and the falsity-membership function,* $F_{P}(x)$*, clearly satisfy condition (2):*

(2) $$0\leq T_{P}(x),I_{P}(x),F_{P}(x)\leq 1,\;0\leq T_{P}(x)+I_{P}(x)+F_{P}(x)\leq 3.$$

For an SVNS, P in X, we call the triplet $\left(T_{P}(x),I_{P}(x),F_{P}(x)\right)$ its single-valued neutrosophic value (SVNV), simply denoted $x=(T_{x},I_{x},F_{x})$ for computational convenience.

**Definition** **2** [17,21]. *Let* $x=(T_{x},I_{x},F_{x})$ *and* $y=(T_{y},I_{y},F_{y})$ *be two SVNVs. Then*

*(1)*  $x⊕y=(T_{x}+T_{y}-T_{x}*T_{y},I_{x}*T_{y},F_{x}*F_{y});$
*(2)*  $\lambda x=(1-{\left(1-T_{x}\right)}^{\lambda },{\left(I_{x}\right)}^{\lambda },{\left(F_{x}\right)}^{\lambda }),$λ>0;
*(3)*  $x^{\lambda }=({\left(T_{x}\right)}^{\lambda },1-{\left(1-I_{x}\right)}^{\lambda },1-{\left(1-F_{x}\right)}^{\lambda })$*,* λ>0.

### 2.2. The Linguistic Set

Let $S=\left\{s_{\alpha }|\alpha =1,...,l\right\}$ be a finite and totally ordered discrete term set with the odd value, l, where $s_{\alpha }$ denotes a possible value for a linguistic variable. For instance, if l=7, then a linguistic term set S could be described as follows:

(3) $$S=\{s_{1},s_{2},s_{3},s_{4},s_{5},s_{6},s_{7}\}\;=\;\{extremely\;poor,\;very\;poor,poor,\;fair,\;good,\;very\;good,\;extremely\;good\}.$$

Any linguistic variables, $s_{i}$ and $s_{j}$, in S must satisfy rules (1)–(4) [47]:

(1) $Neg(s_{i})=s_{-i}$;
(2) $s_{i}\leq s_{j}\Leftrightarrow i\leq j$;
(3) $\max(s_{i},s_{j})=s_{j}$, if i≤j;
(4) $\min(s_{i},s_{j})=s_{j}$, if i≤j.

To prevent information loss in an aggregation process, the discrete term set S shall be stretched to a continuous term set $\bar{S}=\left\{s_{\alpha }|\alpha \in R\right\}$. Any two linguistic variables, $s_{\alpha },s_{\beta }\in \bar{S}$, satisfy following operational laws [48]:

(1) $s_{\alpha }⊕s_{\beta }=s_{\alpha +\beta }$;
(2) $\mu s_{\alpha }=s_{\mu \alpha }$, μ≥0;
(3) $s_{\alpha }/s_{\beta }=s_{\alpha /\beta }$.

### 2.3. The Single-Valued Neutrosophic Linguistic Set (SVNLS)

**Definition** **3** [34]. *Given* X*, a finite universe set, an SVNLS,* P*, in* X *can be defined as in (4):*

(4) $$P=\left\{\langle x,[s_{\theta (x)},(T_{P}(x),I_{P}(x),F_{P}(x))]\rangle |x\in X\right\},$$

*where* $s_{\theta (x)}\in \bar{S}$*, the truth-membership function,* $T_{P}(x)$*, the indeterminacy- membership function,* $I_{P}(x)$*, and the falsity-membership function,* $F_{P}(x)$ *satisfy condition (5):*

(5) $$0\leq T_{P}(x),I_{P}(x),F_{P}(x)\leq 1,\;0\leq T_{P}(x)+I_{P}(x)+F_{P}(x)\leq 3.$$

For an SVNLS, P, in X, the 4-tuple $\langle s_{\theta (x)},(T_{P}(x),I_{P}(x),F_{P}(x))\rangle$ is named an SVNLN, simply denoted $x=\langle s_{\theta (x)},(T_{x},I_{x},F_{x})\rangle$ for computational convenience.

**Definition** **4** [34]. *Let* $x_{i}=\langle s_{\theta (x_{i})},(T_{x_{i}},I_{x_{i}},F_{x_{i}})\rangle (i=1,2)$ *be two SVNLNs. Then*

*(1)*  $x_{1}⊕x_{2}=\langle s_{\theta (x_{1})+\theta (x_{2})},(T_{x_{1}}+T_{x_{2}}-T_{x_{1}}*T_{x_{2}},I_{x_{1}}*T_{x_{2}},F_{x_{1}}*F_{x_{2}})\rangle ;$
*(2)*  $\lambda x_{1}=\langle s_{\lambda \theta (x_{1})},(1-{\left(1-T_{x_{1}}\right)}^{\lambda },{\left(I_{x_{1}}\right)}^{\lambda },{\left(F_{x_{1}}\right)}^{\lambda })\rangle ,$λ>0;
*(3)*  $x_{{}^{1}}^{\lambda }=\langle s_{\theta^{\lambda }(x_{1})},({\left(T_{x_{1}}\right)}^{\lambda },1-{\left(1-I_{x_{1}}\right)}^{\lambda },1-{\left(1-F_{x_{1}}\right)}^{\lambda })\rangle$*,* λ>0.

**Definition** **5** [34]. *Let* $x_{i}=\langle s_{\theta (x_{i})},(T_{x_{i}},I_{x_{i}},F_{x_{i}})\rangle (i=1,2)$ *be two SVNLNs. Their distance measure is defined as in (6):*

(6) $$d(x_{1},x_{2})={\left[{\left|s_{\theta (x_{1})}T_{x_{1}}-s_{\theta (x_{2})}T_{x_{2}}\right|}^{\mu }+{\left|s_{\theta (x_{1})}I_{x_{1}}-s_{\theta (x_{2})}I_{x_{2}}\right|}^{\mu }+{\left|s_{\theta (x_{1})}F_{x_{1}}-s_{\theta (x_{2})}F_{x_{2}}\right|}^{\mu }\right]}^{1/\mu }.$$

In particular, Equation (6) reduces to the Hamming distance of SVNLS and the Euclidean distance of SVNLS when μ=1 and μ=2, respectively.

## 3. MADM Based on the SVNLOWAD-TOPSIS Method

### 3.1. Description of the MADM Problem in SVNL Environments

For a given MADM problem under SNVL environments, $A=\{A_{1},...,A_{m}\}$ denotes a set of discrete feasible alternatives, $C=\{C_{1},...,C_{n}\}$ represents a set of attributes and $E=\{e_{1},...,e_{k}\}$ is a set of experts (or DMs) with the weight vector $\omega ={\left\{\omega_{1},...,\omega_{k}\right\}}^{T}$, such that $\sum_{i=1}^{n}\omega_{i}=1$ and $0\leq \omega_{i}\leq 1$. Suppose that the weight vector of the attributes is $v={\left(v_{1},...,v_{n}\right)}^{T}$, satisfying $\sum_{i=1}^{n}v_{i}=1$ and $v_{i}\in [0,1].$ The evaluation, $\alpha_{{}_{ij}}^{\left(k\right)}$, given by the expert, $e_{t}(t=1,...,k)$, on the alternative, $A_{i}(i=1,...,m)$, relative to attribute $C_{j}(j=1,...,n)$ forms the individual decision matrix in (7):

(7) $$\begin{array}{l} \begin{array}{cccc} & \;C_{1} & \cdots & C_{n} \end{array}\\ D^{k}=\begin{array}{c} A_{1}\\ \vdots \\ A_{n} \end{array}\left(\begin{array}{ccc} \alpha_{11}^{\left(k\right)} & \cdots & \alpha_{1n}^{\left(k\right)}\\ \vdots & \ddots & \vdots \\ \alpha_{m1}^{\left(k\right)} & \cdots & \alpha_{mn}^{\left(k\right)} \end{array}\right). \end{array}$$

where $\alpha_{{}_{ij}}^{\left(k\right)}=\langle s_{{}_{\theta (\alpha_{ij})}}^{k},(T_{{}_{\alpha_{ij}}}^{k},I_{{}_{\alpha_{ij}}}^{k},F_{{}_{\alpha_{ij}}}^{k})\rangle$ is represented by an SVNLN, satisfying $s_{{}_{\theta (\alpha_{ij})}}^{k}\in \bar{S}$, $T_{{}_{\alpha_{ij}}}^{k},I_{{}_{\alpha_{ij}}}^{k},F_{{}_{\alpha_{ij}}}^{k}\in [0,1]$ and $0\leq T_{{}_{\alpha_{ij}}}^{k}+I_{{}_{\alpha_{ij}}}^{k}+F_{{}_{\alpha_{ij}}}^{k}\leq 3$.

### 3.2. The SVNL TOPSIS Method Proposed by Ye 

The classic TOPSIS proposed by Hwang and Yoon [39] has been considered a popular method for handling the MADM problems with precise numbers. This technique assesses alternatives (and selects the best one) based on the distance from the negative ideal solution (NIS) to the positive ideal solution (PIS). The most suitable choice(s) should meet conditions of having the least distance to the PIS as well as the greatest distance to the NIS. Ye [34] extended the classical TOPSIS method to accommodate the SVNLS situation, and the steps of the extended model can be illustrated as follows.

**Step 1.** Normalize individual decision matrices:

In practice, both benefit attributes and cost attributes may exist in MADM problems. Let B and S be the collections of benefit attributes and cost attributes, respectively. Then, we have the conversion rules in (8):

(8) $$\{\begin{array}{c} \begin{array}{ccc} r_{{}_{ij}}^{\left(k\right)}=\alpha_{{}_{ij}}^{\left(k\right)}=\langle s_{{}_{\theta (\alpha_{ij})}}^{k},(T_{{}_{\alpha_{ij}}}^{k},I_{{}_{\alpha_{ij}}}^{k},F_{{}_{\alpha_{ij}}}^{k})\rangle , & \mathrm{for} & j\in B, \end{array}\\ \begin{array}{ccc} r_{{}_{ij}}^{\left(k\right)}=\langle s_{{}_{l-\theta (\alpha_{ij})}}^{k},(T_{{}_{\alpha_{ij}}}^{k},I_{{}_{\alpha_{ij}}}^{k},F_{{}_{\alpha_{ij}}}^{k})\rangle , & \mathrm{for} & j\in S. \end{array} \end{array}$$

Thus, the standardized decision information, $R^{k}={\left(r_{{}_{ij}}^{\left(k\right)}\right)}_{m\times n}$, is established as in (9):

(9) $$R^{k}={\left(r_{{}_{ij}}^{\left(k\right)}\right)}_{m\times n}=\left(\begin{array}{ccc} r_{11}^{\left(k\right)} & \cdots & r_{1n}^{\left(k\right)}\\ \vdots & \ddots & \vdots \\ r_{m1}^{\left(k\right)} & \cdots & r_{mn}^{\left(k\right)} \end{array}\right).$$

**Step 2.** Construct the collective matrix:

All opinions of the individual DMs are aggregated into a group opinion:

(10) $$R={\left(r_{{}_{ij}}^{}\right)}_{m\times n}=\left(\begin{array}{ccc} r_{11}^{} & \cdots & r_{1n}^{}\\ \vdots & \ddots & \vdots \\ r_{m1}^{} & \cdots & r_{mn}^{} \end{array}\right).$$

where $r_{{}_{ij}}^{}=\sum_{k=1}^{t}\omega_{k}r_{{}_{ij}}^{\left(k\right)}$.

**Step 3.** Establish the weighted SVNL decision information:

The weighted SVNL decision matrix, Y, is formed as shown in (11), using the operational laws given in Definition 2 above:

(11) $$Y={\left(y_{{}_{ij}}^{}\right)}_{m\times n}=\left(\begin{array}{ccc} v_{1}r_{11}^{} & \cdots & v_{n}r_{1n}^{}\\ \vdots & \ddots & \vdots \\ v_{1}r_{m1}^{} & \cdots & v_{n}r_{mn}^{} \end{array}\right).$$

**Step 4.** Calculate the distance measures between the alternatives, $A_{i}(i=1,...,m)$, and the PIS, $A_{{}^{j}}^{+}$/NIS $A_{{}^{j}}^{-}$:

(12) $$\{\begin{array}{c} D(A_{i},A^{+})=\sum_{j=1}^{n}d(y_{ij},y_{{}^{j}}^{+})\\ D(A_{i},A^{-})=\sum_{j=1}^{n}d(y_{ij},y_{{}^{j}}^{-}) \end{array}\;\mathrm{for}\;1,...,m.$$

where PIS $A^{+}=(y_{{}^{1}}^{+},...,y_{{}^{m}}^{+})$ and the NIS $A^{-}=(y_{{}^{1}}^{-},...,y_{{}^{m}}^{-})$ are defined as in (13):

(13) $$\{\begin{array}{c} y_{{}^{i}}^{+}=\langle s_{l},(1,0,0)\rangle \\ y_{{}^{i}}^{-}=\langle s_{1},(0,1,1)\rangle \end{array}\;\mathrm{for}\;i=1,...,m.$$

**Step 5.** Compute the relative closeness coefficient, $C(A_{i})$, for the alternatives, $A_{i}(i=1,...,m)$, according to the formula in (14):

(14) $$C(A_{i})=\frac{D(A_{i},A^{-})}{D(A_{i},A^{+})+D(A_{i},A^{-})}.$$

**Step 6.** Order the alternatives and identify the optimal one(s) in accordance with the decreasing values, $C_{i}$, obtained from Step 5.

In summary, the TOPSIS developed by Ye [34] is an effective and simple technique for handling MADM problems under an SVLN environment. However, his method cannot embody the experts’ attitudes, an important factor that needs to be accounted for in some real problems. Aiming to overcome this drawback, we develop a revised SVLN TOPSIS, which enables the model to take into account experts’ attitudinal characters.

### 3.3. The Proposed SVNLOWAD-TOPSIS Model

The ordered weighted averaging (OWA) operator developed by Yager [49] is important in most aggregation techniques and has been analyzed, applied and generalized by a number of researchers [38,50,51,52,53,54,55]. The most distinctive advantage of the OWA operator is its ordering mechanism for the argument under consideration, which enables the integration of the complex attitudinal character of the experts in the decision-making process. Recently, the application of the OWA in distance measure has become a fruitful research issue and has achieved numerous accomplishments, such as the OWA distance (OWAD) [56], the Bonferroni OWAD [57], the continuous OWAD [58], the cloud OWAD [59] and the induced OWAD [60,61]. Given the desirable properties of the OWA operator, a SVNL OWA distance (SVNLOWAD) measure, is proposed in the following text.

**Definition** **6.** *Let*$x_{j},x_{j}^{\prime }$*(*j=1,...,n*) be the two collections of SVNLNs. If*

(15) $$SVNLOWAD\left(\left(x_{1},x_{1}^{\prime }),...,(x_{n},x_{n}^{\prime }\right)\right)=\sum_{j=1}^{n}w_{j}d(x_{j},x_{j}^{\prime }),$$

*then the SVNLOWAD is called the single-value neutrosophic linguistic ordered weighted averaging distance operator, where*$d(x_{j},x_{j}^{\prime })$*denotes the j-th largest distance among the distances*$d(x_{i},x_{i}^{\prime })(i=1,2,...,n)$*, which is the individual distance between SVNLVs*$x_{i}$*and*$x_{i}^{\prime }$*.*$w={\left(w_{1},...,w^{n}\right)}^{T}$*is an associated weight vector of the SVNLOWAD operator, meeting*$\sum_{j=1}^{n}w_{j}=1$*and*$w_{j}\in [0,1]$.

Similar to the OWA operator, the SVNLOWAD operator yields an output according to the attitudinal character (or degree of orness) of the DMs. The attitudinal character formula is expressed in (16):

(16) $$\alpha \left(W\right)=\sum_{j=1}^{n}w_{j}\left(\frac{n-j}{n-1}\right).$$

It can be seen that α(W)∈[0,1]. In addition, the closer to the top weight W is located, the closer α is to 1. In a decision-making progress, the degree of orness can be used to represent the DMs’ attitudinal character degrees of optimism or pessimism.

It is straightforward to prove that the SVNLOWAD operator is idempotent, monotonic, bounded and commutative. Moreover, by signing different values for the weight vector in the SVNLOWAD operator, it is possible to obtain a series of the SVNL distance measures. Therefore, DMs have more choice in selecting cases according to their particular interests or the specific problem being considered. For example,

- The normalized SVNL distance (NSVNLD) is formed if $w={\left(\frac{1}{n},...,\frac{1}{n}\right)}^{T}$.
- The maximum SVNL weighted distance (MaxSVNLD) is obtained when $w={\left(1,0\mathrm{...},0\right)}^{T}$.
- The minimum SVNL weighted distance (MinSVNLD) is obtained when $w={\left(0,...,0,1\right)}^{T}$.
- In general, we have a class of step SVNLOWAD operators if $w_{k}=1$ and $w_{j}=0$(j≠k).
- The median SVNLOWAD is found if $w_{\left(n+1\right)/2}=1$ when n is odd, and $w_{j}=0$, j=1,...,(n−1)/2,(n+3)/2,...,n. When n is even, we let $w_{n/2}=w_{(n/2)+1}=0.5$.
- Following the thrust of recent research [50,52,61,62,63,64], one can explore other uses for the SVNLOWAD operator.

On the basis of the SVNLOWAD measure, we now propose a SVNLOWAD-TOPSIS approach which is able to embed the attitudes of MDs in the decision process. This method includes the following steps:

**Steps 1–3.** These are the same as Steps 1–3 in the description in Section 3.2.

**Step 4.** For each alternative, $A_{i}$, the SVNLOWAD is computed for the PIS, $A^{+}$, and the NIS, $A^{-}$, by using Equation (15):

(17) $$SVNLOWAD(A_{i},A^{+})=\sum_{j=1}^{n}w_{j}\dot{d}(y_{ij},y_{{}^{j}}^{+}),\;i=1,...,m$$

(18) $$SVNLOWAD(A_{i},A^{-})=\sum_{j=1}^{n}w_{j}\dot{d}(y_{ij},y_{{}^{j}}^{-}),\;1,...,m$$

where $\dot{d}(y_{ij},y_{{}^{j}}^{+})$ and $\dot{d}(y_{ij},y_{{}^{j}}^{-})$ are the j-th largest values of $d(y_{ij},y_{{}^{j}}^{+})$ and $d(y_{ij},y_{{}^{j}}^{-})$, respectively.

**Step 5**. In Ye’s TOPSIS method, the relative closeness coefficient, $C_{i}$, determined by Equation (14) is used to rank alternatives. However, some authors have pointed out that, in some cases, the relative closeness cannot reach the goal that the desired solution should simultaneously have the least distance from the PIS and the greatest distance from the NIS [20,52]. Thus, following an idea proposed in references [20,52], in (19)–(21), we introduce a revised relative closeness coefficient, $C^{\prime }(A_{i})$, used to measure the degree to which the alternatives, $A_{i}$ (i=1,...,m), are close to the PIS as well as far away from the NIS, congruently:

(19) $$C^{\prime }(A_{i})=\frac{SVNLOWAD(A_{i},A^{-})}{SVNLOWAD_{\max}(A_{i},A^{-})}-\frac{SVNLOWAD(A_{i},A^{+})}{SVNLOWAD_{\min}(A_{i},A^{+})},$$

where

(20) $$SVNLOWAD_{\max}(A_{i},A^{-})=\underset{1\leq i\leq m}{\max}SVNLOWAD(A_{i},A^{-}),$$

and

(21) $$SVNLOWAD_{\min}(A_{i},A^{+})=\underset{1\leq i\leq m}{\min}SVNLOWAD(A_{i},A^{+}).$$

It is clear that $C^{\prime }(A_{i})\leq 0$ (i=1,...,m), and the bigger the value of $C^{\prime }(A_{i})$ is, the better the alternative, $A_{i}$, is. Moreover, if an alternative, $A^{*}$, meets the conditions $SVNLOWAD(A^{*},A^{-})=SVNLOWAD_{\max}(A^{*},A^{-})$ and $SVNLOWAD(A^{*},A^{+})=SVNLOWAD_{\min}(A^{*},A^{+})$, then $C^{\prime }(A^{*})=0$, and the alternative, $A^{*}$, is the most suitable candidate, as it has minimum distance to the PIS and the maximum distance from the NIS.

**Step 6**. Rank and identify the most desirable alternative(s) based on the decreasing closeness coefficient $C^{\prime }(A_{i})$ obtained using Equation (19).

**Remark** **1.** *To present a complete picture of the aggregation results for a decision analysis, we can consider the different cases of the SVNLOWAD that are proposed in* *Section 4* *to calculate distance measures in Step 3. Then, we can obtain a parameterized family of SVNLOWAD-TOPSIS methods, such as the NSVNLD-TOPSIS, the MaxSVNLD-TOPSIS, the MinSVNLD-TOPSIS, the WSVNLD-TOPSIS and the Step SVNLOWAD-TOPSIS methods.*

## 4. An Illustrative Example

In recent years, global economic development and public health have been threatened by increased carbon emissions, which has prompted companies and governments around the world to attempt to stimulate and increase investments in low-carbon and green economics. In this decision-making process, a key stage is to choose a suitable green supplier in the low-carbon supply chain. More importantly, the process involves various factors involving uncertain information, all of which have to be considered and evaluated simultaneously. Therefore, green supplier selection is a highly ambiguous and complex decision process. To date, several authors have investigated methods for low-carbon selection in such uncertain situations. Lin and Wang [65] and Tong and Wang [66] explored low-carbon supplier selection problems based on the linguistic and IFS MADM approaches, respectively. Liu et al. [67] developed a novel aggregation method for selecting a low-carbon supplier in an intuitionistic linguistic (IL) environment. However, since the linguistic set, IFS and IL, can be viewed as special cases of SVNLS, it is more suitable to utilize the SVNLS to describe the uncertainty in the selection of green and low-carbon supplier problems. To address this important research, we use this section to develop a low-carbon selection example by using the proposed SVNLOWAD-TOPISIS method.

With the help of practitioners and managers in low-carbon supply chain management, three experts are invited to assess and select an appropriated low-carbon supplier as a manufacturer from among four potential suppliers, $A_{i}$(i=1,2,3,4), in accordance with the following four attributes: low-carbon technology ($C_{1}$), cost ($C_{2}$), risk factor ($C_{3}$) and capacity ($C_{4}$) (adapted from [65]). The evaluation provided by the experts against the above-given four attributes is formed into SVNL decision information under the linguistic term set S= {$s_{1}$ = extremely poor, $s_{2}$ = very poor, $s_{3}$ = poor, $s_{4}$ = fair, $s_{5}$ = good, $s_{6}$ = very good, $s_{7}$ = extremely good}, as shown in Table 1, Table 2 and Table 3.

Assume that the weight vectors of the attributes and the DMs are $v={\left(0.20,0.15,0.25,0.4\right)}^{T}$ and $\omega ={\left(0.37,0.33,0.3\right)}^{T}$, respectively. Considering the available information, we employ the proposed approach to select the most suitable choice(s). The main steps are summarized as follows:

**Step 1.** In this problem, since $C_{2}$ and $C_{3}$ are the cost attributes, $\alpha_{{}_{ij}}^{\left(k\right)}=\langle s_{{}_{\theta (\alpha_{ij})}}^{k},(T_{{}_{\alpha_{ij}}}^{k},I_{{}_{\alpha_{ij}}}^{k},F_{{}_{\alpha_{ij}}}^{k})\rangle (j=2,3)$ are converted to the following formula by Equation (8), as in (21):

(22) $$r_{{}_{ij}}^{\left(k\right)}=\langle s_{{}_{7-\theta (\alpha_{ij})}}^{k},(T_{{}_{\alpha_{ij}}}^{k},I_{{}_{\alpha_{ij}}}^{k},F_{{}_{\alpha_{ij}}}^{k})\rangle ,i=1,2,3,4;j=2,3;k=1,2,3.$$

Then, the standardized SVNL decision matrices are established in Table 4, Table 5 and Table 6.

**Step 2**. Calculate the collective opinion and get the collective SVNL decision matrix, which is shown in Table 7:

**Step 3.** Yield the weighted collective SVNL matrix based on SVNL operational rules. The result is shown in Table 8:

**Step 4.** To incorporate their complex attitudes, the DMs determine the weight vector of the OWA operator: $W={\left(0.25,0.30,0.35,0.10\right)}^{T}$. Then, we employ Equations (17) and (18) (assuming p=1) to calculate the $SVNLOWAD(A_{i},A^{+})$ and $SVNLOWAD(A_{i},A^{-})$ measures between the alternative, $A_{i}$, and the PIS, $A^{+}$, and the NIS, $A^{+}$:

$$SVNLOWAD(A_{1},A^{+})=8.223,\;SVNLOWAD(A_{2},A^{+})=8.003,\;SVNLOWAD(A_{3},A^{+})=8.526,\;\;SVNLOWAD(A_{4},A^{+})=7.823,\;SVNLOWAD(A_{1},A^{-})=0.980,\;SVNLOWAD(A_{2},A^{-})=1.106,\;\;SVNLOWAD(A_{3},A^{-})=0.584,\;SVNLOWAD(A_{4},A^{-})=1.467.$$

**Step 5**. Utilize Equation (19) to compute the revised relative coefficients:

$$C^{\prime }(A_{1})=-0.383,\;C^{\prime }(A_{2})=-0.269,\;C^{\prime }(A_{3})=-0.602,\;C^{\prime }(A_{4})=0.$$

**Step 6**. Order all the alternatives according to the closeness, $C^{\prime }(A_{i})$, of each alternative, $A_{i}$. This yields

$$A_{4}≻A_{2}≻A_{1}≻A_{3}.$$

Therefore, the most desirable alternative is $A_{4}$, which coincides with the result obtained by the application of the method proposed by Ye [34]. However, as one can note, the values of the relative coefficients for potential alternatives are different from Ye’s results, which are listed in Table 9.

It is possible to investigate further how the particular cases of the SVNLOWAD-TOPSIS have an impact on the aggregation results. We consider the MaxSVNLD-TOPSIS, the MinSVNLD-TOPSIS, the NSVNLD-TOPSIS, the WSVNLD-TOPSIS and the Step SVNLOWAD (k=2)-TOPSIS methods in this example. The results are given in Table 10 and Table 11.

It is easy to see that application of different instances of the SVNLOWAD-TOPSIS renders different orderings of the alternatives.

In summary, a comparison of the SVNLOWAD-TOPSIS instances with the Ye’s method [34] confirms that the salient feature of the SVNLOWAD-TOPSIS developments is the capability of SVNLOWAD-TOPSIS to take into account the specific interests of the experts relative to the particular practical problem at hand. This model is very flexible, as it can provide more specific cases for DMs to choose from using the different families of the operators.

## 5. Conclusions

In this study, we presented an OWA distance-based TOPSIS method for green supplier selection under a single-valued neutrosophic linguistic environment. The main contributions of this paper can be summarized as follows: (1) Great progress has been achieved in single-valued neutrosophic linguistic distance measure methodology. To effectively measure single-valued neutrosophic linguistic information, we introduced the SVNLOWAD operator, which enables us to fuse the DMs’ attitudinal characters during the aggregation process. (2) We have proposed a novel SVNLOWAD-TOPSIS method for green supplier selection. The outstanding merit of the method is its ability to fuse the optimism or pessimism degrees of DMs. Moreover, it allows for thorough viewing of the decision process, as the decision-makers can choose different values of interest and thereby render many different scenarios. A revised relative coefficient has been proposed to rank the potential alternatives. Therefore, this method provides a more general and more accurate result in comparison with existing methods. (3) A case concerning green supplier selection was presented to demonstrate the calculation process and the feasibility of the proposed method. A comparison with existing methods illustrates that the proposed model can be expected to achieve more realistic and accurate results in single-valued neutrosophic linguistic situations. Thus, this paper offers a significant contribution in regard to the development of MADM frameworks for green supplier selection problems.

In regard to future research, we propose to offer further extensions of the SVNLOWAD-TOPSIS method by employing additional aggregation techniques, e.g., unified aggregation operators, induced variables and a refined neutrosophic set [68]. Other empirical applications of this method will also be considered in future works.

## Figures and Tables

**Table 1:** Single-valued neutrosophic linguistic (SVNL) decision matrix $D^{1}$.

Alternatives	$C_{1}$	$C_{2}$	$C_{3}$	$C_{4}$
$A_{1}$	$\langle s_{{}_{5}}^{\left(1\right)},(0.4,0.2,0.3)\rangle$	$\langle s_{{}_{2}}^{\left(1\right)},(0.4,0.2,0.3)\rangle$	$\langle s_{4}^{\left(1\right)},(0.3,0.2,0.5)\rangle$	$\langle s_{4}^{\left(1\right)},(0.5,0.3,0.3)\rangle$
$A_{2}$	$\langle s_{{}_{6}}^{\left(1\right)},(0.6,0.1,0.2)\rangle$	$\langle s_{{}_{2}}^{\left(1\right)},(0.6,0.1,0.2)\rangle$	$\langle s_{{}_{3}}^{\left(1\right)},(0.5,0.2,0.2)\rangle$	$\langle s_{3}^{\left(1\right)},(0.6,0.2,0.4)\rangle$
$A_{3}$	$\langle s_{{}_{4}}^{\left(1\right)},(0.3,0.2,0.3)\rangle$	$\langle s_{{}_{3}}^{\left(1\right)},(0.5,0.2,0.3)\rangle$	$\langle s_{{}_{4}}^{\left(1\right)},(0.5,0.3,0.1)\rangle$	$\langle s_{5}^{\left(1\right)},(0.3,0.5,0.2)\rangle$
$A_{4}$	$\langle s_{{}_{5}}^{\left(1\right)},(0.7,0.0,0.1)\rangle$	$\langle s_{3}^{\left(1\right)},(0.6,0.1,0.2)\rangle$	$\langle s_{{}_{4}}^{\left(1\right)},(0.3,0.1,0.2)\rangle$	$\langle s_{6}^{\left(1\right)},(0.6,0.1,0.2)\rangle$

**Table 2:** SVNL decision matrix $D^{2}$.

Alternatives	$C_{1}$	$C_{2}$	$C_{3}$	$C_{4}$
$A_{1}$	$\langle s_{{}_{5}}^{\left(2\right)},(0.4,0.3,0.4)\rangle$	$\langle s_{{}_{1}}^{\left(2\right)},(0.5,0.1,0.2)\rangle$	$\langle s_{2}^{\left(2\right)},(0.3,0.1,0.6)\rangle$	$\langle s_{3}^{\left(2\right)},(0.7,0.1,0.1)\rangle$
$A_{2}$	$\langle s_{{}_{6}}^{\left(2\right)},(0.7,0.2,0.3)\rangle$	$\langle s_{{}_{1}}^{\left(2\right)},(0.7,0.2,0.3)\rangle$	$\langle s_{2}^{\left(2\right)},(0.6,0.2,0.2)\rangle$	$\langle s_{4}^{\left(2\right)},(0.5,0.4,0.2)\rangle$
$A_{3}$	$\langle s_{{}_{6}}^{\left(2\right)},(0.4,0.2,0.4)\rangle$	$\langle s_{{}_{1}}^{\left(2\right)},(0.6,0.3,0.4)\rangle$	$\langle s_{3}^{\left(2\right)},(0.6,0.1,0.3)\rangle$	$\langle s_{5}^{\left(2\right)},(0.4,0.4,0.1)\rangle$
$A_{4}$	$\langle s_{{}_{4}}^{\left(2\right)},(0.8,0.1,0.2)\rangle$	$\langle s_{{}_{2}}^{\left(2\right)},(0.7,0.2,0.3)\rangle$	$\langle s_{3}^{\left(2\right)},(0.4,0.2,0.2)\rangle$	$\langle s_{{}_{6}}^{\left(2\right)},(0.6,0.3,0.3)\rangle$

**Table 3:** SVNL decision matrix $D^{3}$.

Alternatives	$C_{1}$	$C_{2}$	$C_{3}$	$C_{4}$
$A_{1}$	$\langle s_{{}_{6}}^{\left(3\right)},(0.5,0.2,0.3)\rangle$	$\langle s_{1}^{\left(3\right)},(0.6,0.2,0.4)\rangle$	$\langle s_{2}^{\left(3\right)},(0.2,0.1,0.6)\rangle$	$\langle s_{4}^{\left(3\right)},(0.5,0.2,0.3)\rangle$
$A_{2}$	$\langle s_{{}_{5}}^{\left(3\right)},(0.5,0.2,0.3)\rangle$	$\langle s_{3}^{\left(3\right)},(0.7,0.2,0.2)\rangle$	$\langle s_{2}^{\left(3\right)},(0.7,0.2,0.1)\rangle$	$\langle s_{6}^{\left(3\right)},(0.4,0.6,0.2)\rangle$
$A_{3}$	$\langle s_{{}_{6}}^{\left(3\right)},(0.5,0.1,0.3)\rangle$	$\langle s_{2}^{\left(3\right)},(0.6,0.1,0.3)\rangle$	$\langle s_{3}^{\left(3\right)},(0.6,0.2,0.1)\rangle$	$\langle s_{4}^{\left(3\right)},(0.3,0.6,0.2)\rangle$
$A_{4}$	$\langle s_{{}_{4}}^{\left(3\right)},(0.6,0.1,0.2)\rangle$	$\langle s_{3}^{\left(3\right)},(0.5,0.2,0.2)\rangle$	$\langle s_{4}^{\left(3\right)},(0.4,0.1,0.1)\rangle$	$\langle s_{5}^{\left(3\right)},(0.7,0.2,0.1)\rangle$

**Table 4:** Standardized SVNL decision matrix $R^{1}$.

Alternatives	$C_{1}$	$C_{2}$	$C_{3}$	$C_{4}$
$A_{1}$	$\langle s_{{}_{5}}^{\left(1\right)},(0.4,0.2,0.3)\rangle$	$\langle s_{{}_{5}}^{\left(1\right)},(0.4,0.2,0.3)\rangle$	$\langle s_{3}^{\left(1\right)},(0.3,0.2,0.5)\rangle$	$\langle s_{4}^{\left(1\right)},(0.5,0.3,0.3)\rangle$
$A_{2}$	$\langle s_{{}_{6}}^{\left(1\right)},(0.6,0.1,0.2)\rangle$	$\langle s_{{}_{5}}^{\left(1\right)},(0.6,0.1,0.2)\rangle$	$\langle s_{{}_{4}}^{\left(1\right)},(0.5,0.2,0.2)\rangle$	$\langle s_{3}^{\left(1\right)},(0.6,0.2,0.4)\rangle$
$A_{3}$	$\langle s_{{}_{4}}^{\left(1\right)},(0.3,0.2,0.3)\rangle$	$\langle s_{{}_{4}}^{\left(1\right)},(0.5,0.2,0.3)\rangle$	$\langle s_{{}_{3}}^{\left(1\right)},(0.5,0.3,0.1)\rangle$	$\langle s_{5}^{\left(1\right)},(0.3,0.5,0.2)\rangle$
$A_{4}$	$\langle s_{{}_{5}}^{\left(1\right)},(0.7,0.0,0.1)\rangle$	$\langle s_{4}^{\left(1\right)},(0.6,0.1,0.2)\rangle$	$\langle s_{{}_{3}}^{\left(1\right)},(0.3,0.1,0.2)\rangle$	$\langle s_{6}^{\left(1\right)},(0.6,0.1,0.2)\rangle$

**Table 5:** Standardized SVNL decision matrix $R^{2}$.

Alternatives	$C_{1}$	$C_{2}$	$C_{3}$	$C_{4}$
$A_{1}$	$\langle s_{{}_{5}}^{\left(2\right)},(0.4,0.3,0.4)\rangle$	$\langle s_{{}_{6}}^{\left(2\right)},(0.5,0.1,0.2)\rangle$	$\langle s_{5}^{\left(2\right)},(0.3,0.1,0.6)\rangle$	$\langle s_{3}^{\left(2\right)},(0.7,0.1,0.1)\rangle$
$A_{2}$	$\langle s_{{}_{6}}^{\left(2\right)},(0.7,0.2,0.3)\rangle$	$\langle s_{{}_{6}}^{\left(2\right)},(0.7,0.2,0.3)\rangle$	$\langle s_{5}^{\left(2\right)},(0.6,0.2,0.2)\rangle$	$\langle s_{4}^{\left(2\right)},(0.5,0.4,0.2)\rangle$
$A_{3}$	$\langle s_{{}_{6}}^{\left(2\right)},(0.4,0.2,0.4)\rangle$	$\langle s_{{}_{6}}^{\left(2\right)},(0.6,0.3,0.4)\rangle$	$\langle s_{4}^{\left(2\right)},(0.6,0.1,0.3)\rangle$	$\langle s_{5}^{\left(2\right)},(0.4,0.4,0.1)\rangle$
$A_{4}$	$\langle s_{{}_{4}}^{\left(2\right)},(0.8,0.1,0.2)\rangle$	$\langle s_{{}_{5}}^{\left(2\right)},(0.7,0.2,0.3)\rangle$	$\langle s_{4}^{\left(2\right)},(0.4,0.2,0.2)\rangle$	$\langle s_{{}_{6}}^{\left(2\right)},(0.6,0.3,0.3)\rangle$

**Table 6:** Standardized SVNL decision matrix $R^{3}$.

Alternatives	$C_{1}$	$C_{2}$	$C_{3}$	$C_{4}$
$A_{1}$	$\langle s_{{}_{6}}^{\left(3\right)},(0.5,0.2,0.3)\rangle$	$\langle s_{6}^{\left(3\right)},(0.6,0.2,0.4)\rangle$	$\langle s_{5}^{\left(3\right)},(0.2,0.1,0.6)\rangle$	$\langle s_{4}^{\left(3\right)},(0.5,0.2,0.3)\rangle$
$A_{2}$	$\langle s_{{}_{5}}^{\left(3\right)},(0.5,0.2,0.3)\rangle$	$\langle s_{4}^{\left(3\right)},(0.7,0.2,0.2)\rangle$	$\langle s_{5}^{\left(3\right)},(0.7,0.2,0.1)\rangle$	$\langle s_{6}^{\left(3\right)},(0.4,0.6,0.2)\rangle$
$A_{3}$	$\langle s_{{}_{6}}^{\left(3\right)},(0.5,0.1,0.3)\rangle$	$\langle s_{5}^{\left(3\right)},(0.6,0.1,0.3)\rangle$	$\langle s_{4}^{\left(3\right)},(0.6,0.2,0.1)\rangle$	$\langle s_{4}^{\left(3\right)},(0.3,0.6,0.2)\rangle$
$A_{4}$	$\langle s_{{}_{4}}^{\left(3\right)},(0.6,0.1,0.2)\rangle$	$\langle s_{4}^{\left(3\right)},(0.5,0.2,0.2)\rangle$	$\langle s_{3}^{\left(3\right)},(0.4,0.1,0.1)\rangle$	$\langle s_{5}^{\left(3\right)},(0.7,0.2,0.1)\rangle$

**Table 7:** Collective SVNL decision matrix R.

Alternatives	$C_{1}$	$C_{2}$	$C_{3}$	$C_{4}$
$A_{1}$	$\langle s_{{}_{5.30}}^{},(0.432,0.229,0.330)\rangle$	$\langle s_{5.63}^{},(0.450,0.159,0.286)\rangle$	$\langle s_{2.37}^{},(0.271,0.129,0.561)\rangle$	$\langle s_{3.67}^{},(0.578,0.185,0.209)\rangle$
$A_{2}$	$\langle s_{{}_{5.70}}^{},(0.611,0.155,0.258)\rangle$	$\langle s_{4.70}^{},(0.666,0.155,0.229)\rangle$	$\langle s_{2.37}^{},(0.602,0.200,0.162)\rangle$	$\langle s_{4.23}^{},(0.514,0.350,0.258)\rangle$
$A_{3}$	$\langle s_{{}_{5.26}}^{},(0.399,0.163,0.330)\rangle$	$\langle s_{4.96}^{},(0.566,0.186,0.330)\rangle$	$\langle s_{3.37}^{},(0.566,0.185,0.144)\rangle$	$\langle s_{4.70}^{},(0.335,0.491,0.159)\rangle$
$A_{4}$	$\langle s_{{}_{4.37}}^{},(0.714,0.000,0.155)\rangle$	$\langle s_{4.33}^{},(0.611,0.155,0.229)\rangle$	$\langle s_{3.67}^{},(0.365,0.128,0.163)\rangle$	$\langle s_{5.70}^{},(0.633,0.180,0.186)\rangle$

**Table 8:** Weighted collective SVNL decision matrix Y.

Alternatives	$C_{1}$	$C_{2}$	$C_{3}$	$C_{4}$
$A_{1}$	$\langle s_{{}_{1.06}}^{},(0.107,0.745,0.801)\rangle$	$\langle s_{1.126}^{},(0.113,0.692,0.779)\rangle$	$\langle s_{0.474}^{},(0.061,0.664,0.891)\rangle$	$\langle s_{0.734}^{},(0.158,0.713,0.731)\rangle$
$A_{2}$	$\langle s_{{}_{0.855}}^{},(0.132,0.756,0.816)\rangle$	$\langle s_{0.705}^{},(0.152,0.756,0.801)\rangle$	$\langle s_{0.356}^{},(0.129,0.786,0.761)\rangle$	$\langle s_{0.634}^{},(0.103,0.854,0.816)\rangle$
$A_{3}$	$\langle s_{{}_{1.315}}^{},(0.119,0.635,0.758)\rangle$	$\langle s_{1.240}^{},(0.188,0.657,0.758)\rangle$	$\langle s_{0.843}^{},(0.188,0.656,0.616)\rangle$	$\langle s_{1.175}^{},(0.097,0.837,0.632)\rangle$
$A_{4}$	$\langle s_{{}_{1.748}}^{},(0.394,0.000,0.474)\rangle$	$\langle s_{1.732}^{},(0.315,0.474,0.555)\rangle$	$\langle s_{0.146}^{},(0.053,0.484,0.555)\rangle$	$\langle s_{2.280}^{},(0.330,0.500,0.510)\rangle$

**Table 9:** Results obtained by the Ye’s method [34].

Alternatives	$D(A_{i},A^{+})$	$D(A_{i},A^{-})$	$C(A_{i})$	Ranking
$A_{1}$	32.527	3.754	0.103	3
$A_{2}$	31.720	4.280	0.119	2
$A_{3}$	33.722	2.278	0.063	4
$A_{4}$	31.071	5.534	0.151	1

**Table 10:** Closeness coefficients, $C^{\prime }(A_{i})$, obtained under particular cases of the SVNLOWAD-TOPSIS approach.

Alternatives	MaxSVNLD-TOPSIS	MinSVNLD-TOPSIS	NSVNLD-TOPSIS	WSVNLD-TOPSIS	Step SVNLOWAD-TOPSIS (k=2)
$A_{1}$	−0.227	−0.437	−0.401	−0.469	−0.370
$A_{2}$	−0.196	−0.051	−0.247	−0.155	−0.456
$A_{3}$	−0.470	−0.651	−0.583	−0.016	−0.819
$A_{4}$	−0.039	−0.008	0	0	0.030

**Table 11:** Ordering of the alternatives based on particular cases of the SVNLOWAD-TOPSIS approach.

Particular Cases	Ordering
MaxSVNLD-TOPSIS	$A_{4}≻A_{2}≻A_{1}≻A_{3}$
MinSVNLD-TOPSIS	$A_{4}≻A_{2}≻A_{1}≻A_{3}$
NSVNLD-TOPSIS	$A_{4}≻A_{2}≻A_{1}≻A_{3}$
WSVNLD-TOPSIS	$A_{4}≻A_{3}≻A_{2}≻A_{1}$
Step SVNLOWAD-TOPSIS (k=2)	$A_{4}≻A_{1}≻A_{2}≻A_{3}$
